# "Reactivity to Stimuli” Is a Temperamental Factor Contributing to Canine Aggression

**DOI:** 10.1371/journal.pone.0100767

**Published:** 2014-06-27

**Authors:** Sayaka Arata, Yukari Takeuchi, Mai Inoue, Yuji Mori

**Affiliations:** 1 Department of Animal Resource Sciences, The University of Tokyo, Tokyo, Japan; 2 Claims Service Department, Anicom Insurance Inc., Tokyo, Japan; University of Sydney, Australia

## Abstract

Canine aggression is one of the most frequent problems in veterinary behavioral medicine, which in severe cases may result in relinquishment or euthanasia. As it is important to reveal underlying factors of aggression for both treatment and prevention, we recently developed a questionnaire on aggression and temperamental traits and found that “reactivity to stimuli” was associated with aggression toward owners, children, strangers, and other dogs of the Shiba Inu breed. In order to examine whether these associations were consistent in other breeds, we asked the owners of insured dogs of Anicom Insurance Inc. to complete our questionnaire. The top 17 contracted breeds were included. The questionnaire consisted of dogs' general information, four items related to aggression toward owners, children, strangers, and other dogs, and 20 other behavioral items. Aggression-related and behavioral items were rated on a five-point frequency scale. Valid responses (n = 5610) from owners of dogs aged 1 through 10 years were collected. Factor analyses on 18 behavioral items (response rate over 95%) extracted five largely consistent factors in 14 breeds: “sociability with humans,” “fear of sounds,” “chase proneness,” “reactivity to stimuli,” and “avoidance of aversive events.” By stepwise multiple regression analyses, using the Schwartz's Bayesian information criterion (BIC) method with aggression points as objective variables and general information and temperamental factor points as explanatory variables, “reactivity to stimuli,” i.e., physical reactivity to sudden movement or sound at home, was shown to be significantly associated with owner-directed aggression in 13 breeds, child-directed aggression in eight breeds, stranger-directed aggression in nine breeds, and dog-directed aggression in five breeds. These results suggest that “reactivity to stimuli” is simultaneously involved in several types of aggression. Therefore, it would be worth taking “reactivity to stimuli” into account in the treatment and prevention of canine aggression.

## Introduction

Canine aggression is one of the most severe problems in veterinary behavioral medicine and a frequent reason for consultation [1], [2]. As dogs' sharp teeth can cause considerable damage, severe cases may sometimes result in relinquishment or euthanasia [2]–[5]. In clinical practice, aggression can be classified by its target (owner-, stranger-, or dog-directed) or motivation (fear-, territorial-, or possessiveness-related, and so on) [6]. The motivation can be different for owner-, stranger-, or dog-directed aggression; however, the co-occurrence of types of aggression has been reported [2], [7]. Therefore, it is thought that there are factors that are common to several types of aggression and factors that are specific to each type.

Behavioral traits in dogs are often assessed using a behavior test (including test batteries and an observational test) and a questionnaire survey (categorized as ratings of individual dogs) [8]. Regardless of the assessment method, it should be reliable, valid, and feasible [8], [9]. Although a behavior test is considered to be more objective, it is not always possible to replicate various situations or stimuli from daily life, particularly for aggression [5], [10]–[13], and it usually takes time to complete, resulting in limited validity and poorer feasibility [14]. On the other hand, a questionnaire survey rated by a person familiar with the dog, i.e., an owner, family member, or trainer, is generally feasible and can include several situations at a time [15]. One of the most widely used assessments is the Canine Behavioral Assessment and Research Questionnaire (C-BARQ) [14], [16], which has been shown to have high reliability and validity [17].

Recently, we conducted a questionnaire survey on the Shiba Inu breed [18] using four aggression items (owner-, child-, stranger-, and dog-directed aggression) and 14 behavioral items generated based on the C-BARQ [14], [16]. When we compared aggression items and temperamental factors extracted by the factor analysis of behavioral items, one of the temperamental factors, “reactivity to stimuli,” was commonly significantly associated with the four types of aggression. Therefore, it is suggested that “reactivity to stimuli” might be one of the temperamental traits that predisposes Shiba Inus to aggressive behavior regardless of targets [18]. However, as behavioral traits are well known to differ among dog breeds [4], [5], [19]–[22], we still do not know whether these associations are consistent in other breeds.

In this study, we conducted a questionnaire survey on over 5000 dogs of 17 breeds using the 14 behavioral items that appeared in our previous report [18] and six new items relating to general fear and anxiety. We examined associations of four types of aggression (owner-, child-, stranger-, and dog-directed aggression) with behavioral traits and general information, and tested our hypothesis that “reactivity to stimuli” was associated with several types of aggression in other dog breeds.

## Materials and Methods

### Behavioral assessment

The questionnaire consisted of general information on the dogs including breed, date of birth, age, sex, age of neutering, source of acquisition (pet shop, breeder, or other), age at acquisition, and housing condition (indoor, outdoor, or both); four items related to aggression towards owners, children, strangers and other dogs; and 20 other behavioral items. The aggression-related items were listed on a separate page from the behavioral items. As shown in Table 1, the 14 behavioral items were the willingness to approach humans, fearful response to noises, chasing behavior toward creatures, sudden movements toward indoor stimuli that were consistent with our previous paper, and six other behavioral items on fearful or avoidance behavior that were included in this study. Owners were asked to score their dog's responses within the last 3 months for aggression and behavioral items using five-point frequency scales (5 =  always, 4 =  often, 3 =  sometimes, 2 =  occasionally, and 1 =  never) or as “unknown” (owners were instructed to answer “unknown” if the situation described in the question had not been observed or the dog's response could not be recalled clearly).

**Table 1:** The four aggression items and 20 behavioral items included in the questionnaire.

Item	Description
*Aggression*	
Owner-directed aggression	Does the dog growl aggressively at or bite household members?
Child-directed aggression	Does the dog growl aggressively at or bite children outside of the household?
Stranger-directed aggression	Does the dog growl aggressively at or bite unfamiliar men/women?
Dog-directed aggression	Does the dog growl aggressively at or bite unfamiliar dogs?
*Behavioral trait*	
Q1: Sociability with men	Does the dog willingly approach unfamiliar men while out on a walk?
Q2: Sociability with women	Does the dog willingly approach unfamiliar women while out on a walk?
Q3: Sociability with children	Does the dog willingly approach unfamiliar children while out on a walk?
Q4: Fear of heavy traffic	Does the dog show any behaviors such as bending lower, flattening his/her ears, trembling, or trying to get behind in heavy traffic?
Q5: Fear of thunder	Does the dog show any behaviors such as bending lower, flattening his/her ears, trembling, or trying to get behind during thunderstorms, firework displays, or similar events?
Q6: Fear of engine noises	Does the dog show any behaviors such as bending lower, flattening his/her ears, or trying to get behind in response to sudden or loud engine noises from automobiles or motorcycles?
Q7: Chase proneness to cats	Does the dog pounce on or chase cats?
Q8: Chase proneness to birds	Does the dog pounce on or chase pigeons, crows, or other birds?
Q9: Chase proneness to other creatures	Does the dog pounce on or chase worms, lizards, frogs, or other moving small animals?
Q10: Chase proneness to falling leaves	Does the dog pounce on or chase leaves or other wind-blown objects?
Q11: Reactivity to movement of hands	Does the dog pounce on or stare at movements such as passing by or moving hands in front of it while it is resting?
Q12: Reactivity to movement of feet	Does the dog pounce on or stare at movements such as swinging feet under the table?
Q13: Reactivity to clattering dishes	Does the dog bark or come to investigate in response to sudden or loud noises of dishes, pans, or pots being dropped?
Q14: Reactivity to phone ringing	Does the dog bark or come to investigate when the telephone rings?
Q15: Anxiety at unfamiliar places	Does the dog freeze or tremble at novel and/or unfamiliar places (e.g., on a trip, or taking a walk using an unusual route)?
Q16: Anxiety under unfamiliar situations	Does the dog freeze or tremble at novel and/or unfamiliar situation (e.g., riding on a bicycle or elevator for the first time, or in a crowd)?
Q17: Fear of unusual things	Does the dog try to avoid, freeze, or tremble at novel and/or unfamiliar objects (e.g., plastic bags, carts, carriers, skateboards, or wheel chairs)?
Q18: Avoidance of aversive places	Does the dog try to avoid or freeze when heading towards a place that relates to a previous aversive experience (e.g., veterinary hospital)?
Q19: Avoidance of examination table	Does the dog try to avoid, freeze, or tremble on the examination table at a veterinary hospital?
Q20: Fear of darkness and/or heights	Does the dog freeze or tremble with darkness and/or heights?

The questionnaire items are listed in the order in which they appeared on the actual questionnaire sheet.

The aggression items were on a separate sheet from the behavioral trait items.

The questions were answered using a frequency scale [5 =  always (100%), 4 =  often (99–61%), 3 =  sometimes (60–40%), 2 =  occasionally (39–1%), 1 =  never (0%)] or “unknown”.

### Subjects

The questionnaire survey was announced to the owners of dogs insured by Anicom Insurance Inc. by e-mail. We targeted the top 17 contracted breeds with more than 3000 insured dogs (in descending order of frequency, the Miniature Dachshund, Chihuahua, Toy Poodle, Shiba Inu, Yorkshire Terrier, Welsh Corgi Pembroke, Papillon, Pomeranian, Shih Tzu, Miniature Schnauzer, Labrador Retriever, French Bulldog, Cavalier King Charles Spaniel, Golden Retriever, Maltese, Pug, and Jack Russell Terrier), and the announcement was sent to a total of 87,537 owners with an informed consent to use the data for academic research. We asked the owners to complete voluntarily our questionnaire on the web between June 5^th^ and July 5^th^ in 2012.

### Data analysis

The answers on the questionnaire excepting personal information of owners such as owner's name, e-mail address and insurance number were given to the authors belonging to the University of Tokyo in order not to be leaked the information out of the company, and further analyses were done only by these authors. Dogs aged 1–10 years were used for the analyses. When a certain dog was assessed more than once, the first answer was adopted. The behavioral items “chase proneness to other creatures” and “fear of darkness or heights” were excluded because of low response rates (90.3% and 94.0%, respectively) and factor analyses were conducted on the other 18 items in each breed. To extract common factors in as many breeds as possible, factor analyses were repeated taking behavioral items in and out. Factor extraction was performed by the principal factor method, and the Varimax rotation was used for orthogonal transformation. The extracted factors were determined using the eigenvalue criterion (i.e., the eigenvalue for the last extracted factor was greater than 1.0). The questionnaire items for which the absolute loading on a factor was 0.4 or more were considered to belong to the factor. To assess the internal consistency of the factor, Cronbach's α reliability coefficients were calculated for the items belonging to each factor. The factor points were calculated by averaging the raw scores of the items constituting each factor. Mean scores of each type of aggression and mean factor points of each temperamental factor were calculated for each of the 14 breeds that showed consistent results in factor analyses, and were then applied to cluster analysis with Ward's method. Finally, stepwise multiple regression analyses with the Schwartz's Bayesian information criterion (BIC) method were performed using each aggression point as an objective variable and the dog's general information [age, sex (male/female), neutered (yes/no), source of acquisition (pet shop, breeder, or other), age at acquisition, and housing condition (indoor, outdoor, or both)] and extracted factor points as explanatory variables on the 14 breeds. When the number of applicable dogs in a certain category of the factors “source of acquisition” or “housing condition” was less than six, the category was excluded from the analyses. Breed differences in aggression points and factor points were examined by ANOVA. These analyses were performed using StatView 5J for Windows (SAS Institute, Cary, NC) and JMP 9.0 (SAS Institute, Cary, NC). The significance level was set to 5%.

## Results

### Data analysis

Valid responses (n = 5610) from owners of dogs aged 1 through 10 years were collected from 1299 Toy Poodles, 1144 Miniature Dachshunds, 964 Chihuahuas, 505 Shiba Inus, 302 Welsh Corgi Pembrokes, 272 Papillons, 270 Miniature Schnauzers, 267 Golden Retrievers, 262 Yorkshire Terriers, 249 Labrador Retrievers, 249 French Bulldogs, 218 Pomeranians, 215 Cavalier King Charles Spaniels, 209 Shih Tzus, 146 Jack Russell Terriers, 136 Malteses, and 124 Pugs.

### Factor analyses

Factor analyses of 14 items extracted five largely-consistent factors in 14 breeds excepting the Welsh Corgi Pembroke, Shih Tzu, and Pug: “sociability with humans,” “fear of sounds,” “chase-proneness,” “reactivity to stimuli,” and “avoidance of aversive events.” Table 2 shows the result of a factor analysis using the 14 breeds as a whole, and the results for each of the 17 breeds appear in Table S1. Cronbach's α coefficients exceeded 0.7 in “sociability with humans,” “fear of sounds,” “chase-proneness,” and “avoidance of aversive events,” and 0.592 in “reactivity to stimuli.”

**Table 2:** Factor analysis of behavioral items using 14 breeds (n = 4922).

	Sociability with humans	Fear of sounds	Chase proneness	Reactivity to stimuli	Avoidance of aversive events
Sociability with men	**0.934**	−0.045	0.096	0.019	−0.068
Sociability with women	**0.941**	−0.040	0.091	0.032	−0.055
Sociability with children	**0.862**	−0.042	0.142	0.029	−0.063
Fear of engine noises	−0.069	**0.776**	−0.037	0.148	0.142
Fear of thunder	−0.021	**0.805**	0.021	−0.004	0.071
Fear of heavy traffic	−0.028	**0.885**	0.005	0.105	0.082
Chase proneness to cats	0.067	−0.036	**0.854**	0.021	0.044
Chase proneness to birds	0.079	−0.024	**0.892**	0.043	0.047
Chase proneness to falling leaves	0.164	0.049	**0.688**	0.188	−0.025
Reactivity to movement of hands	0.089	0.109	0.157	**0.617**	0.067
Reactivity to clattering dishes	0.003	0.060	0.116	**0.785**	0.115
Reactivity to phone ringing	−0.025	0.048	−0.041	**0.773**	0.015
Avoidance of aversive place	−0.077	0.165	0.029	0.095	**0.877**
Avoidance of examination table	−0.085	0.112	0.034	0.099	**0.888**
Eigenvalue	3.053	2.749	1.714	1.325	1.218
Contribution ratio (%)	21.8	19.6	12.2	9.5	8.7
Cronbach's α	0.914	0.776	0.772	0.592	0.775

The questionnaire items for which the absolute loading on a factor was 0.4 or more are shown in boldface.

### Breed difference on scores of aggression and temperamental factors

**Figure 1:**
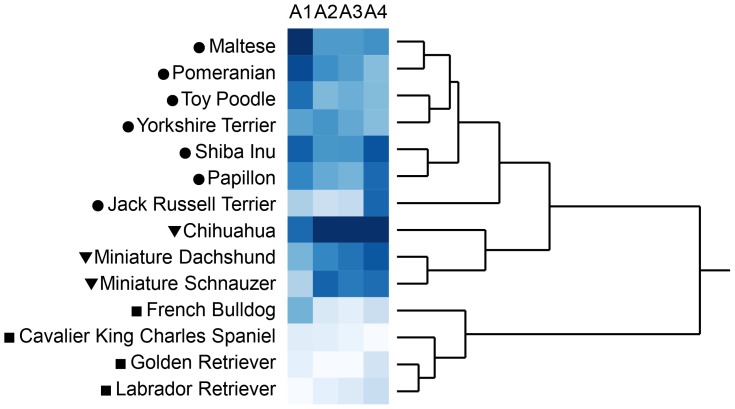
Cluster analysis on scores of four types of aggression in 14 dog breeds. Cluster analysis with Ward's method was conducted on aggression scores of 14 breeds. A1–4 is aggression toward owner, child, stranger, and dog, respectively. Degree of aggression scores are shown in color spectrum (from white to blue). Tree diagram is drawn with distance scale. Symbols ahead of breed names represent the groups when the number of clusters is set to three.

**Figure 2:**
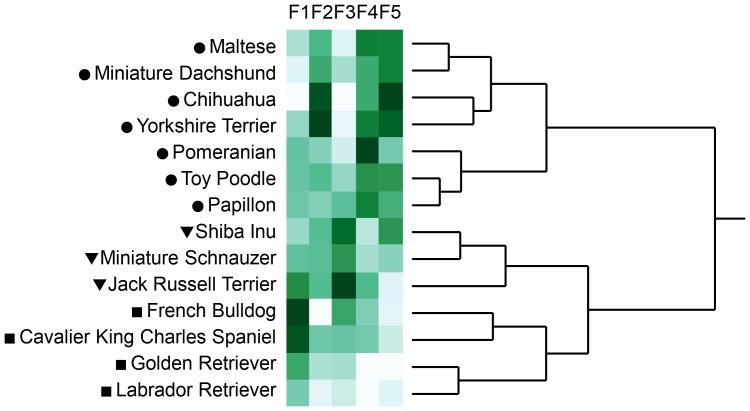
Cluster analysis on points of five temperamental factors in 14 dog breeds. Cluster analysis with Ward's method was conducted on temperament factor points of 14 breeds. F1–5 is “sociability with humans,” “fear of sounds,” “chase proneness,” “reactivity to stimuli,” and “avoidance of aversive events,” respectively. Degree of aggression points are shown in color spectrum (from white to green). Tree diagram is drawn with distance scale. Symbols ahead of breed names represent the groups when the number of clusters is set to three.

Aggression scores were significantly different among the 14 breeds (p<0.0001 for all four types of aggression, Table S2). Results of cluster analysis on aggression scores are shown in Figure 1. When the 14 breeds were categorized into three groups according to the distance among the clusters, the four breeds (French Bulldog, Cavalier King Charles Spaniel, Golden Retriever, and Labrador Retriever) were generally low in aggression, the three breeds (Chihuahua, Miniature Dachshund, and Miniature Schnauzer) were highly aggressive toward unfamiliar people and dogs, and the other seven breeds (Maltese, Pomeranian, Toy Poodle, Yorkshire Terrier, Shiba Inu, Papillon, and Jack Russell Terrier) were highly aggressive toward owner and moderately aggressive to others. When we classified dogs that scored 1 as non-aggressive and dogs that scored 2 to 5 as aggressive, aggressive dogs accounted for 6.5–51.9% in owner-directed aggression, 5.3–53.6% in child-directed aggression, 14.0–59.8% in stranger-directed aggression, and 28.2–68.8% in dog-directed aggression, depending on breed (Figures S1–S4). Factor points were significantly different among 14 breeds (p<0.001 for all five factors, Table S3). Results of cluster analysis on factor points are shown in Figure 2. When the 14 breeds were categorized into three groups according to the distance among the clusters, the seven breeds (Maltese, Miniature Dachshund, Chihuahua, Yorkshire Terrier, Pomeranian, Toy Poodle, and Papillon) had lower points for “sociability with humans” and “chase-proneness” and higher points for “fear of sounds,” “reactivity to stimuli,” and “avoidance of aversive events”, the three breeds (Shiba Inu, Miniature Schnauzer, and Jack Russell Terrier) had higher points for “chase proneness” and moderate points for other factors, and the four breeds (French Bulldog, Cavalier King Charles Spaniel, Golden Retriever, and Labrador Retriever) had higher points for “sociability with humans,” lower points for “fear of sounds,” “reactivity to stimuli,” and “avoidance of aversive events”, and moderate points for “chase proneness”.

### Multiple regression analyses

Table 3–6 shows the results of multiple regression analyses in 14 breeds. The associations that were significant in more than half of the breeds (seven or more breeds) were “sociability with humans” with child-, stranger-, and dog-directed aggression; “chase-proneness” with dog-directed aggression; “reactivity to stimuli” with owner-, child-, and stranger-directed aggression; and “avoidance of aversive events” with child- and stranger-directed aggression. None of the general information was significantly associated with aggression in more than seven breeds.

**Table 3:** Multiple regression analysis on owner-directed aggression.

Breed	R^2^	n	age	age at acquisition	source of acquisition	housing condition	sex	neutured	sociability with humans	fear of sounds	chase proneness	reactivity to stimuli	avoidance of aversive events
					[pet shop/breeder/other]	[indoor/outdoor/both]	[male/female]	[yes/no]					
Maltese	0.097	106										0.312^ b^	
Pomeranian	0.145	178										0.380^ a^	
Shiba Inu	0.081	406										0.284^ a^	
Chihuahua	0.108	805	0.103^ b^							0.090^ c^		0.230^ a^	0.123^ a^
Toy Poodle	0.066	992										0.220^ a^	0.105^ a^
Papillon	0.064	225	0.158^ c^									0.198^ b^	
Yorkshire Terrier	0.117	213					0.170/−0.170^ c^		−0.152^ c^			0.227^ a^	
French Bull dog	0.175	211			0.244/−0.108/−0.136^ b^		0.157/−0.157^ b^					0.313^ a^	
Miniature Dachshund	0.054	982					0.093/−0.093^ b^					0.180^ a^	0.093^ b^
Jack Russell Terrier	-	125											
Miniature Schnauzer	0.053	216										0.231^ a^	
Cavalier King Charles Spaniel	0.034	177										0.184^ c^	
Golden Retriever	0.084	211										0.290^ a^	
Labrador Retriever	0.096	181										0.308^ a^	
N			2	0	1	0	3	0	1	1	0	13	3

Values are coefficient of determination (R^2^), the number of animals (n), and Standard partial regression coefficients of selected variables.

N shows the number of breeds out of 14 in which a certain variable was selected in the multiple regression analyses.

Four explanatory variables (“source of acquisition”, “housing condition”, “sex”, and “neutered”) were applied as nominal variables.

a: p<0.001, b: p<0.01, c: p<0.05.

**Table 4:** Multiple regression analysis on child-directed aggression.

Breed	R^2^	n	age	age at acquisition	source of acquisition	housing condition	sex	neutured	sociability with humans	fear of sounds	chase proneness	reactivity to stimuli	avoidance of aversive events
					[pet shop/breeder/other]	[indoor/outdoor/both]	[male/female]	[yes/no]					
Maltese	0.064	94											0.253 ^c^
Pomeranian	0.049	167											0.222 ^b^
Shiba Inu	0.066	378							−0.151 ^b^			0.241 ^a^	
Chihuahua	0.108	750							−0.176 ^a^		0.097 ^a^	0.174 ^b^	0.113 ^b^
Toy Poodle	0.089	933							−0.241 ^a^		0.135 ^b^	0.115 ^a^	0.101 ^a^
Papillon	0.088	217							−0.197 ^b^			0.224 ^a^	
Yorkshire Terrier	0.208	199							−0.315 ^a^		0.285 ^a^		0.155 ^c^
French Bull dog	0.076	205										0.302 ^a^	
Miniature Dachshund	0.114	930							−0.195 ^a^		0.187 ^a^	0.128 ^a^	0.120 ^a^
Jack Russell Terrier	0.083	118											0.289 ^b^
Miniature Schnauzer	0.083	207							−0.211 ^c^			0.227 ^a^	
Cavalier King Charles Spaniel	0.076	174										0.275 ^a^	
Golden Retriever	0.140	206								0.193 ^b^		0.291 ^a^	
Labrador Retriever	0.055	179									0.234 ^b^		
N			0	0	0	0	0	0	7	1	5	9	7

Values are coefficient of determination (R^2^), the number of animals (n), and Standard partial regression coefficients of selected variables.

N shows the number of breeds out of 14 in which a certain variable was selected in the multiple regression analyses.

Four explanatory variables (“source of acquisition”, “housing condition”, “sex”, and “neutered”) were applied as nominal variables.

a: p<0.001, b: p<0.01, c: p<0.05

**Table 5:** Multiple regression analysis on stranger-directed aggression.

Breed	R^2^	n	age	age at acquisition	source of acquisition	housing condition	sex	neutured	sociability with humans	fear of sounds	chase proneness	reactivity to stimuli	avoidance of aversive events
					[pet shop/breeder/other]	[indoor/outdoor/both]	[male/female]	[yes/no]					
Maltese	0.088	105											0.296^ b^
Pomeranian	0.066	176											0.256^ a^
Shiba Inu	0.113	405				−0.200/0.252/−0.052^ a^			−0.177^ a^			0.179^ a^	
Chihuahua	0.143	780							−0.248^ a^		0.137^ a^	0.194^ a^	0.104^ b^
Toy Poodle	0.110	967							−0.203^ a^		0.115^ a^	0.193^ a^	0.115^ a^
Papillon	0.078	224							−0.280^ a^				
Yorkshire Terrier	0.223	207							−0.340^ a^		0.214^ a^		0.214^ a^
French Bull dog	0.098	211											0.288^ a^
Miniature Dachshund	0.131	963							−0.219^ a^		0.198^ a^	0.125^ a^	0.136^ a^
Jack Russell Terrier	0.138	124										0.186^ c^	0.273^ b^
Miniature Schnauzer	0.069	216							−0.206^ b^			0.197^ b^	
Cavalier King Charles Spaniel	0.096	176										0.310^ a^	
Golden Retriever	0.217	204	−0.165^ c^					−0.174/0.174^ b^	−0.333^ a^		0.183^ b^	0.226^ b^	
Labrador Retriever	0.029	179										0.171^ c^	
N			1	0	0	1	0	1	8	0	5	9	8

Values are coefficient of determination (R^2^), the number of animals (n), and Standard partial regression coefficients of selected variables.

N shows the number of breeds out of 14 in which a certain variable was selected in the multiple regression analyses.

Four explanatory variables (“source of acquisition”, “housing condition”, “sex”, and “neutered”) were applied as nominal variables.

a: p<0.001, b: p<0.01, c: p<0.05.

**Table 6:** Multiple regression analysis on dog-directed aggression.

Breed	R^2^	n	age	age at acquisition	source of acquisition	housing condition	sex	neutured	sociability with humans	fear of sounds	chase proneness	reactivity to stimuli	avoidance of aversive events
					[pet shop/breeder/other]	[indoor/outdoor/both]	[male/female]	[yes/no]					
Maltese	0.127	102									0.356^ a^		
Pomeranian	0.064	174	0.205^ b^								0.180^ c^		
Shiba Inu	0.093	406	0.217^ a^					−0.121/0.121^ c^				0.226^ a^	
Chihuahua	0.129	769	0.106^ b^						−0.197^ a^		0.330^ a^		
Toy Poodle	0.143	972							−0.136^ a^		0.256^ a^	0.128^ a^	0.185^ a^
Papillon	0.155	222	0.198^ b^						−0.219^ b^		0.302^ a^		
Yorkshire Terrier	0.155	208	0.171^ c^						−0.197^ b^		0.321^ a^		
French Bull dog	0.029	210										0.174^ c^	
Miniature Dachshund	0.120	963							−0.090^ b^		0.283^ a^	0.098^ b^	0.104^ a^
Jack Russell Terrier	0.190	125											0.436^ a^
Miniature Schnauzer	0.139	215							−0.227^ a^		0.364^ a^		
Cavalier King Charles Spaniel	0.135	175			0.077/−0.503/0.580^ a^							0.220^ b^	
Golden Retriever	0.172	208					0.146/−0.146^ c^		−0.157^ c^		0.221^ b^		0.279^ a^
Labrador Retriever	0.054	181									0.232^ a^		
N			5	0	1	0	1	1	7	0	10	5	4

Values are coefficient of determination (R^2^), the number of animals (n), and Standard partial regression coefficients of selected variables.

N shows the number of breeds out of 14 in which a certain variable was selected in the multiple regression analyses.

Four explanatory variables (“source of acquisition”, “housing condition”, “sex”, and “neutered”) were applied as nominal variables.

a: p<0.001, b: p<0.01, c: p<0.05.

## Discussionf

**Figure 3:**
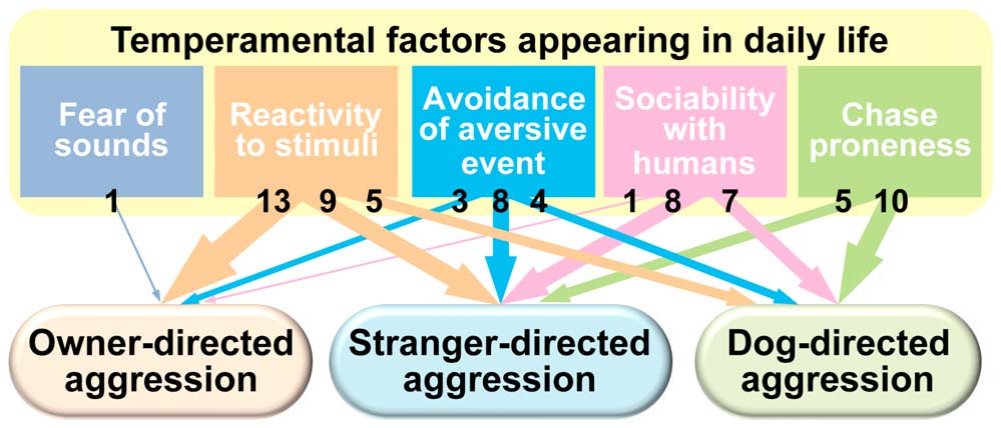
The associations between temperamental factors appearing in daily life and aggression in 14 dog breeds. Multiple regression analyses in each breed revealed associations between five temperamental factors and four types of aggression. The width of arrows and numbers show the number of breeds in which a temperamental factor was significantly associated with a type of aggression.

In this study, we conducted a questionnaire survey on over 5000 dogs of 17 breeds, and a factor analysis using 14 behavioral items consistently extracted five temperamental factors in 14 breeds. Multiple regression analyses revealed that temperamental factors were specifically or commonly associated with each type of aggression (Figure 3).

The questionnaire in this study was based on our previous report [18] with six added fear/anxiety-related items. In 14 out of 17 breeds, factor analysis of the behavioral traits extracted five largely-consistent factors, suggesting that these factors are comparatively stable in dogs. Except for “avoidance of aversive events” consisting of the added items, four factors were identical to ones from our previous study in Shiba Inu [18]. The associations between aggression and temperamental factors in Shiba Inus were replicated; the “reactivity to stimuli” was significantly associated with all of the four types of aggression (Table 3–6). These results support the reliability of our questionnaire survey.

Cluster analysis on aggression scores and factor points (Figure 1 and 2) revealed breed characteristics; for example, the four breeds (French Bulldog, Cavalier King Charles Spaniel, Golden Retriever, and Labrador Retriever) were generally less aggressive, sociable with humans, less fearful, less prone to chasing, and less reactive. On the other hand, Chihuahua and Miniature Dachshund were comparatively aggressive to strangers, less sociable with humans, fearful, and prone to chasing. They are mostly consistent with the previous reports using C-BARQ answered by owners [5] and assessments done by veterinary practitioners [20], suggesting the validity of rating by dog owners in this study.

Regarding the association between aggression and temperamental factors, “reactivity to stimuli” was significantly associated with owner-directed aggression in 13 breeds, child-directed aggression in eight breeds, stranger-directed aggression in nine breeds, and dog-directed aggression in five breeds. Thus, our hypothesis that “reactivity to stimuli” is simultaneously involved in several types of aggression, particularly human-directed aggression, was most verified. Although Shiba Inus are known to have unique characteristics in that they are genetically close to wolves [23] and tend to display higher aggression [20] than Western breeds, it is interesting that Western breeds showed similar associations to those found in our previous study of Shiba Inus [18]. This factor consists of items on physical reactivity to sudden movement or sound inside the house, and dogs with high “reactivity to stimuli” may include two types of dogs; one type is restless and seems to react to almost all the movements and sounds, and the other is generally less active, but overreacts to sudden stimuli, particularly when relaxed or during sleep, as described in Q11–14 of our questionnaire (Table 1). Wright et al., [24] developed a questionnaire assessing impulsivity in dogs, and showed that ‘Aggression & Response to Novelty' factor was positively correlated with impulsivity. Although the terminology is different, both Wright et al. and our present study appear to have examined similar traits.

“Sociability with humans” was significantly associated with child-, stranger-, and dog-directed aggression in seven or more breeds. This trait is a well-studied behavioral phenomenon that shows an association with stranger-directed aggression [5], [14],[17], and the results in this study are consistent with those of previous studies. Similar associations were seen for our new factor “avoidance of aversive events”; however, it is interesting that the associations with child- and stranger-directed aggression were not always consistent for “sociability with humans” and “avoidance of aversive events” (Table 4 and 5). Only “sociability with humans” was selected in the Papillon, Miniature Schnauzer, and Shiba Inu, and only “avoidance of aversive events” was selected in the Jack Russell Terrier, Pomeranian, and Maltese. On the basis of these results, it is considered that the aforementioned three breeds might tend to show aggression due to anxiety and/or inadequate socialization, and the latter three breeds due to specific traumatic experiences.

“Chase proneness” was significantly associated with dog-directed aggression in 10 breeds, and dogs that tend to chase small moving animals or objects were more aggressive toward other dogs. Chasing and chase-proneness have been assessed by the C-BARQ and the Dog Mentality Assessment conducted by the Swedish Working Dog Association, respectively, but none of the studies have shown associations between chasing/chase-proneness and dog-directed aggression [16], [17]. The discrepancy between the previous studies and ours might be associated with the high prevalence of dog-directed aggression in this study; the frequency of aggressive dogs (dogs scoring 2–5) toward other dogs was 55.9% in 14 breeds as a whole (Figure S4). It is important to examine whether this association is characteristic of dogs in Japan or is due to other reasons.

The involvement of “reactivity to stimuli” in aggression is expected to be useful in two areas: veterinary clinical behavior medicine and behavior genetics. There are various ways to treat canine aggression, including management, behavior modification, and medication. As “reactivity to stimuli” was generally associated with human-directed aggression, it is recommended to avoid sudden or unusual manners of approaching or touching a dog for treatment and prevention of aggression. Instead, based on our clinical experience, calling the dog's name before approaching the dog can be effective in avoiding aggression. Genetic research on canine behavior has been gaining prominence [25], and aggression is a well-studied trait [26]–[29] because of its impact on human society and high heritability in the Golden Retriever [26]. We have shown that genetic polymorphism in the glutamate transporter gene, *SLC1A2*, is associated with stranger-directed aggression in the Shiba Inu [27], and suggested that the excitation/inhibition system of neurotransmission might play a role in canine aggression. In general, searching for aggression-related genes is associated with difficulties, since aggression is considered to be the final output derived from various motivations even if it is categorized by its target; e.g., owner-directed aggression can be possessive aggression or fearful aggression. As “reactivity to stimuli” in this study was shown to be an underlying temperamental factor of canine aggression in various breeds, it should bring a more accurate assessment of aggression, which is essential for canine behavioral genetics.

In conclusion, our questionnaire survey on 14 breeds was shown to have reliability and validity, and one of the temperamental factors, “reactivity to stimuli,” was simultaneously associated with several types of aggression in most of the breeds. This factor is suggested to contribute to canine aggression, partially explaining the co-occurrence of several types of aggression. Therefore, “reactivity to stimuli” should be taken into account in the treatment and prevention of canine aggression, and will be valuable for understanding the genetic basis of canine aggression.

## Supporting Information

Figure S1 **The prevalence of owner-directed aggression in 14 dog breeds.** The proportion of answers (1 =  never, 2 =  occasionally, 3 =  sometimes, 4 =  often, and 5 =  always) for owner-directed aggression is shown for each breed in the order of aggressiveness.
(DOC)

Figure S2 **The prevalence of child-directed aggression in 14 dog breeds.** The proportion of answers (1 =  never, 2 =  occasionally, 3 =  sometimes, 4 =  often, and 5 =  always) for child-directed aggression is shown for each breed in the order of aggressiveness.
(DOC)

Figure S3 **The prevalence of stranger-directed aggression in 14 dog breeds.** The proportion of answers (1 =  never, 2 =  occasionally, 3 =  sometimes, 4 =  often, and 5 =  always) for stranger-directed aggression is shown for each breed in the order of aggressiveness.
(DOC)

Figure S4 **The prevalence of dog-directed aggression in 14 dog breeds.** The proportion of answers (1 =  never, 2 =  occasionally, 3 =  sometimes, 4 =  often, and 5 =  always) for dog-directed aggression is shown for each breed in the order of aggressiveness.
(DOC)

Table S1 Factor analysis of behavioral items in each breed.
(DOC)

Table S2 Scores of four types of aggression in 14 breeds.
(DOC)

Table S3 Points of five temperamental factors in 14 breeds.
(DOC)
