# Supplementary material for: "Reactivity to Stimuli” Is a Temperamental Factor Contributing to Canine Aggression
Source: PLoS One. 2014 Jun 27;9(6):e100767. doi: 10.1371/journal.pone.0100767 (PMC4074066; doi:10.1371/journal.pone.0100767)
Supplement: Figure S1 — The prevalence of owner-directed aggression in 14 dog breeds. The proportion of answers (1 = never, 2 = occasionally, 3 = sometimes, 4 = often, and 5 = always) for owner-directed aggression is shown for each breed in the order of aggressiveness. (DOC) [file pone.0100767.s001.doc]

**Figure S1:** The prevalence of owner-directed aggression in 14 dog breeds.

The proportion of answers (1 = never, 2 = occasionally, 3 = sometimes, 4 = often, and 5 = always) for owner-directed aggression is shown for each breed in the order of aggressiveness.
