# Supplementary material for: "Reactivity to Stimuli” Is a Temperamental Factor Contributing to Canine Aggression
Source: PLoS One. 2014 Jun 27;9(6):e100767. doi: 10.1371/journal.pone.0100767 (PMC4074066; doi:10.1371/journal.pone.0100767)
Supplement: Table S1 — Factor analysis of behavioral items in each breed. (DOC) [file pone.0100767.s005.doc]

**Table S1A:** Factor analysis of behavioral items on Toy Poodle (n = 864)

|  | Factor 1 | Factor 2 | Factor 3 | Factor 4 | Factor 5 |
| --- | --- | --- | --- | --- | --- |
| Sociability with men | **0.930** | -0.042 | 0.099 | -0.054 | 0.030 |
| Sociability with women | **0.937** | -0.034 | 0.084 | -0.061 | 0.024 |
| Sociability with children | **0.852** | -0.017 | 0.160 | -0.098 | 0.032 |
| Fear of engine noises | -0.021 | **0.793** | -0.042 | 0.106 | 0.144 |
| Fear of thunder | 0.002 | **0.831** | 0.039 | 0.028 | 0.013 |
| Fear of heavy traffic | -0.050 | **0.881** | -0.020 | 0.068 | 0.086 |
| Chase proneness to cats | 0.105 | -0.033 | **0.875** | 0.037 | 0.038 |
| Chase proneness to birds | 0.078 | -0.058 | **0.899** | 0.022 | 0.045 |
| Chase proneness to falling leaves | 0.139 | 0.076 | **0.698** | -0.066 | 0.158 |
| Reactivity to movement of hands | 0.137 | 0.095 | 0.240 | 0.062 | **0.574** |
| Reactivity to clattering dishes | 0.005 | 0.075 | 0.048 | 0.131 | **0.785** |
| Reactivity to phone ringing | -0.046 | 0.059 | -0.011 | -0.045 | **0.778** |
| Avoidance of aversive place | -0.084 | 0.145 | -0.008 | **0.887** | 0.080 |
| Avoidance of examination table | -0.115 | 0.045 | 0.002 | **0.904** | 0.055 |
| Eigenvalue | 3.124 | 2.590 | 1.783 | 1.378 | 1.274 |
| Contribution ratio (%) | 22.3 | 18.5 | 12.7 | 9.8 | 9.1 |
| Cronbach's α | 0.904 | 0.793 | 0.794 | 0.780 | 0.559 |

The questionnaire items for which the absolute loading on a factor was 0.4 or more are shown in boldface.

**Table S1B:** Factor analysis of behavioral items on Miniature Dachshund (n = 880)

|  | Factor 1 | Factor 2 | Factor 3 | Factor 4 | Factor 5 |
| --- | --- | --- | --- | --- | --- |
| Sociability with men | **0.934** | 0.024 | -0.049 | -0.079 | 0.075 |
| Sociability with women | **0.943** | 0.016 | -0.052 | -0.065 | 0.057 |
| Sociability with children | **0.873** | 0.019 | -0.063 | -0.022 | 0.108 |
| Fear of engine noises | -0.120 | 0.112 | **0.770** | 0.104 | -0.013 |
| Fear of thunder | 0.025 | -0.074 | **0.807** | 0.093 | 0.009 |
| Fear of heavy traffic | -0.060 | 0.104 | **0.873** | 0.035 | 0.026 |
| Chase proneness to cats | 0.039 | 0.012 | -0.054 | 0.163 | **0.829** |
| Chase proneness to birds | 0.078 | 0.066 | 0.006 | 0.053 | **0.888** |
| Chase proneness to falling leaves | 0.127 | 0.263 | 0.082 | -0.155 | **0.647** |
| Reactivity to movement of hands | 0.058 | **0.577** | 0.009 | -0.002 | 0.197 |
| Reactivity to clattering dishes | -0.019 | **0.791** | 0.075 | 0.066 | 0.144 |
| Reactivity to phone ringing | 0.000 | **0.776** | 0.041 | 0.049 | -0.080 |
| Avoidance of aversive place | -0.004 | 0.071 | 0.149 | **0.884** | 0.007 |
| Avoidance of examination table | -0.139 | 0.037 | 0.076 | **0.883** | 0.074 |
| Eigenvalue | 2.954 | 2.544 | 1.743 | 1.419 | 1.301 |
| Contribution ratio (%) | 21.1 | 18.2 | 12.5 | 10.1 | 9.3 |
| Cronbach's α | 0.913 | 0.557 | 0.762 | 0.774 | 0.737 |

The questionnaire items for which the absolute loading on a factor was 0.4 or more are shown in boldface.

**Table S1C:** Factor analysis of behavioral items on Chihuahua (n = 693)

|  | Factor 1 | Factor 2 | Factor 3 | Factor 4 | Factor 5 |
| --- | --- | --- | --- | --- | --- |
| Sociability with men | **0.930** | -0.071 | 0.074 | -0.002 | -0.069 |
| Sociability with women | **0.937** | -0.072 | 0.078 | 0.010 | -0.078 |
| Sociability with children | **0.865** | -0.059 | 0.123 | 0.016 | -0.050 |
| Fear of engine noises | -0.068 | **0.819** | -0.042 | 0.090 | 0.103 |
| Fear of thunder | -0.084 | **0.796** | 0.033 | 0.070 | 0.130 |
| Fear of heavy traffic | -0.048 | **0.887** | 0.010 | 0.081 | 0.036 |
| Chase proneness to cats | 0.047 | -0.055 | **0.866** | 0.060 | 0.087 |
| Chase proneness to birds | 0.053 | -0.059 | **0.902** | 0.006 | 0.074 |
| Chase proneness to falling leaves | 0.185 | 0.140 | **0.636** | 0.220 | -0.039 |
| Reactivity to movement of hands | 0.087 | 0.290 | 0.091 | **0.561** | 0.088 |
| Reactivity to clattering dishes | 0.021 | 0.044 | 0.142 | **0.811** | 0.109 |
| Reactivity to phone ringing | -0.073 | -0.013 | 0.019 | **0.806** | -0.049 |
| Avoidance of aversive place | -0.102 | 0.144 | 0.060 | 0.060 | **0.885** |
| Avoidance of examination table | -0.070 | 0.108 | 0.055 | 0.060 | **0.899** |
| Eigenvalue | 3.088 | 2.752 | 1.763 | 1.401 | 1.224 |
| Contribution ratio (%) | 22.1 | 19.7 | 12.6 | 10.0 | 8.7 |
| Cronbach's α | 0.915 | 0.811 | 0.764 | 0.590 | 0.785 |

The questionnaire items for which the absolute loading on a factor was 0.4 or more are shown in boldface.

**Table S1D:** Factor analysis of behavioral items on Shiba Inu (n = 369)

|  | Factor 1 | Factor 2 | Factor 3 | Factor 4 | Factor 5 |
| --- | --- | --- | --- | --- | --- |
| Sociability with men | **0.930** | 0.004 | 0.109 | -0.042 | 0.125 |
| Sociability with women | **0.930** | 0.014 | 0.082 | -0.042 | 0.107 |
| Sociability with children | **0.832** | -0.092 | 0.198 | -0.042 | 0.058 |
| Fear of engine noises | -0.028 | **0.760** | 0.027 | 0.083 | 0.116 |
| Fear of thunder | -0.016 | **0.766** | -0.019 | 0.056 | -0.010 |
| Fear of heavy traffic | -0.019 | **0.877** | -0.006 | 0.129 | 0.064 |
| Chase proneness to cats | 0.115 | 0.013 | **0.845** | -0.001 | 0.014 |
| Chase proneness to birds | 0.108 | 0.043 | **0.882** | 0.073 | 0.109 |
| Chase proneness to falling leaves | 0.151 | -0.059 | **0.684** | -0.052 | 0.274 |
| Reactivity to movement of hands | 0.064 | 0.046 | 0.124 | 0.099 | **0.661** |
| Reactivity to clattering dishes | 0.082 | 0.024 | 0.077 | 0.043 | **0.764** |
| Reactivity to phone ringing | 0.078 | 0.081 | 0.084 | -0.004 | **0.661** |
| Avoidance of aversive place | -0.066 | 0.134 | 0.066 | **0.872** | 0.054 |
| Avoidance of examination table | -0.039 | 0.117 | -0.045 | **0.874** | 0.083 |
| Eigenvalue | 3.253 | 2.437 | 1.593 | 1.272 | 1.161 |
| Contribution ratio (%) | 23.2 | 17.4 | 11.4 | 9.1 | 8.3 |
| Cronbach's α | 0.894 | 0.713 | 0.758 | 0.715 | 0.515 |

The questionnaire items for which the absolute loading on a factor was 0.4 or more are shown in boldface.

**Table S1E:** Factor analysis of behavioral items on Welsh Corgi Pembroke (n = 230)

|  | Factor 1 | Factor 2 | Factor 3 | Factor 4 | Factor 5 |
| --- | --- | --- | --- | --- | --- |
| Sociability with men | 0.077 | **0.932** | 0.037 | -0.022 | -0.045 |
| Sociability with women | 0.055 | **0.945** | 0.048 | 0.045 | 0.017 |
| Sociability with children | 0.033 | **0.803** | 0.031 | 0.158 | -0.157 |
| Fear of engine noises | **0.759** | 0.079 | 0.041 | 0.156 | 0.131 |
| Fear of thunder | **0.733** | 0.054 | 0.074 | -0.062 | 0.168 |
| Fear of heavy traffic | **0.890** | 0.018 | 0.024 | 0.092 | 0.014 |
| Chase proneness to cats | 0.052 | 0.054 | **0.814** | 0.096 | 0.124 |
| Chase proneness to birds | 0.132 | 0.040 | **0.836** | 0.092 | 0.167 |
| Chase proneness to falling leaves | 0.027 | 0.184 | **0.410** | **0.509** | 0.049 |
| Reactivity to movement of hands | 0.148 | -0.048 | 0.064 | **0.753** | -0.212 |
| Reactivity to clattering dishes | -0.009 | 0.111 | 0.004 | **0.733** | 0.317 |
| Reactivity to phone ringing | 0.204 | 0.126 | **-0.431** | 0.342 | 0.395 |
| Avoidance of aversive place | 0.140 | -0.089 | 0.202 | -0.024 | **0.810** |
| Avoidance of examination table | 0.134 | -0.118 | 0.093 | 0.062 | **0.778** |
| Eigenvalue | 3.026 | 2.438 | 1.643 | 1.293 | 1.172 |
| Contribution ratio (%) | 21.6 | 17.4 | 11.7 | 9.2 | 8.4 |
| Cronbach’s α | 0.693 | 0.894 | 0.449 | 0.474 | 0.700 |

The questionnaire items for which the absolute loading on a factor was 0.4 or more are shown in boldface.

**Table S1F:** Factor analysis of behavioral items on Papillon (n = 207)

|  | Factor 1 | Factor 2 | Factor 3 | Factor 4 | Factor 5 |
| --- | --- | --- | --- | --- | --- |
| Sociability with men | **0.931** | 0.027 | 0.079 | -0.032 | 0.028 |
| Sociability with women | **0.935** | -0.027 | 0.154 | -0.026 | 0.001 |
| Sociability with children | **0.870** | 0.082 | 0.165 | -0.018 | -0.012 |
| Fear of engine noises | -0.028 | **0.787** | 0.064 | 0.078 | 0.060 |
| Fear of thunder | 0.029 | **0.759** | -0.017 | 0.111 | 0.044 |
| Fear of heavy traffic | 0.085 | **0.898** | -0.023 | 0.057 | 0.091 |
| Chase proneness to cats | 0.137 | -0.013 | **0.817** | -0.162 | 0.126 |
| Chase proneness to birds | 0.035 | -0.085 | **0.853** | 0.031 | 0.120 |
| Chase proneness to falling leaves | 0.235 | 0.130 | **0.671** | 0.090 | -0.045 |
| Reactivity to movement of hands | 0.197 | 0.099 | -0.156 | -0.188 | **0.707** |
| Reactivity to clattering dishes | -0.073 | 0.006 | 0.206 | 0.165 | **0.727** |
| Reactivity to phone ringing | -0.116 | 0.141 | 0.200 | 0.303 | **0.536** |
| Avoidance of aversive place | 0.014 | 0.072 | 0.015 | **0.896** | 0.011 |
| Avoidance of examination table | -0.060 | 0.166 | -0.070 | **0.854** | 0.140 |
| Eigenvalue | 3.068 | 2.530 | 1.795 | 1.369 | 1.122 |
| Contribution ratio (%) | 21.9 | 18.1 | 12.8 | 9.8 | 8.0 |
| Cronbach's α | 0.916 | 0.738 | 0.738 | 0.784 | 0.389 |

The questionnaire items for which the absolute loading on a factor was 0.4 or more are shown in boldface.

**Table S1G:** Factor analysis of behavioral items on Miniature Schnauzer (n = 195)

|  | Factor 1 | Factor 2 | Factor 3 | Factor 4 | Factor 5 |
| --- | --- | --- | --- | --- | --- |
| Sociability with men | **0.941** | -0.022 | 0.014 | 0.064 | 0.055 |
| Sociability with women | **0.940** | -0.028 | -0.011 | 0.064 | 0.032 |
| Sociability with children | **0.785** | 0.002 | -0.011 | 0.239 | 0.089 |
| Fear of engine noises | -0.025 | **0.716** | -0.090 | -0.021 | 0.245 |
| Fear of thunder | -0.029 | **0.777** | 0.192 | -0.024 | -0.087 |
| Fear of heavy traffic | 0.010 | **0.873** | -0.005 | 0.134 | 0.063 |
| Chase proneness to cats | 0.125 | -0.021 | 0.022 | **0.834** | 0.094 |
| Chase proneness to birds | 0.072 | 0.016 | 0.018 | **0.908** | -0.023 |
| Chase proneness to falling leaves | 0.238 | 0.167 | 0.094 | **0.632** | 0.376 |
| Reactivity to movement of hands | 0.108 | 0.026 | 0.155 | 0.233 | **0.680** |
| Reactivity to clattering dishes | 0.055 | 0.056 | -0.061 | 0.111 | **0.721** |
| Reactivity to phone ringing | -0.006 | 0.080 | 0.064 | -0.074 | **0.714** |
| Avoidance of aversive place | -0.047 | 0.009 | **0.872** | -0.011 | 0.148 |
| Avoidance of examination table | 0.039 | 0.072 | **0.885** | 0.096 | -0.012 |
| Eigenvalue | 3.292 | 2.211 | 1.579 | 1.468 | 1.320 |
| Contribution ratio (%) | 23.5 | 15.8 | 11.3 | 10.5 | 9.4 |
| Cronbach's α | 0.877 | 0.686 | 0.711 | 0.763 | 0.578 |

The questionnaire items for which the absolute loading on a factor was 0.4 or more are shown in boldface.

**Table S1H:** Factor analysis of behavioral items on Golden Retriever (n = 192)

|  | Factor 1 | Factor 2 | Factor 3 | Factor 4 | Factor 5 |
| --- | --- | --- | --- | --- | --- |
| Sociability with men | **0.951** | 0.036 | -0.040 | 0.029 | 0.138 |
| Sociability with women | **0.945** | 0.084 | -0.048 | 0.070 | 0.143 |
| Sociability with children | **0.893** | 0.173 | -0.016 | -0.010 | 0.092 |
| Fear of engine noises | 0.000 | -0.001 | **0.813** | 0.191 | 0.104 |
| Fear of thunder | -0.067 | 0.087 | **0.703** | -0.058 | 0.039 |
| Fear of heavy traffic | -0.010 | 0.060 | **0.868** | 0.127 | 0.036 |
| Chase proneness to cats | -0.009 | **0.831** | 0.067 | 0.104 | 0.057 |
| Chase proneness to birds | 0.120 | **0.823** | -0.045 | 0.238 | 0.072 |
| Chase proneness to falling leaves | 0.259 | **0.664** | 0.201 | -0.076 | 0.279 |
| Reactivity to movement of hands | 0.163 | 0.254 | 0.070 | 0.276 | **0.573** |
| Reactivity to clattering dishes | 0.078 | 0.224 | 0.136 | 0.232 | **0.745** |
| Reactivity to phone ringing | 0.132 | -0.038 | 0.011 | -0.069 | **0.785** |
| Avoidance of aversive place | -0.004 | 0.130 | 0.141 | **0.844** | 0.118 |
| Avoidance of examination table | 0.056 | 0.103 | 0.071 | **0.864** | 0.113 |
| Eigenvalue | 3.834 | 2.476 | 1.518 | 1.245 | 1.072 |
| Contribution ratio (%) | 27.4 | 17.7 | 10.8 | 8.9 | 7.7 |
| Cronbach's α | 0.944 | 0.723 | 0.655 | 0.760 | 0.594 |

The questionnaire items for which the absolute loading on a factor was 0.4 or more are shown in boldface.

**Table S1I:** Factor analysis of behavioral items on Yorkshire Terrier (n = 177)

|  | Factor 1 | Factor 2 | Factor 3 | Factor 4 | Factor 5 |
| --- | --- | --- | --- | --- | --- |
| Sociability with men | -0.013 | **0.943** | 0.043 | -0.009 | -0.104 |
| Sociability with women | 0.049 | **0.949** | -0.017 | -0.005 | -0.034 |
| Sociability with children | 0.041 | **0.870** | 0.150 | -0.013 | -0.108 |
| Fear of engine noises | **0.769** | -0.087 | 0.020 | 0.188 | 0.124 |
| Fear of thunder | **0.747** | 0.117 | -0.126 | -0.115 | 0.174 |
| Fear of heavy traffic | **0.898** | 0.102 | 0.000 | 0.065 | 0.121 |
| Chase proneness to cats | -0.054 | 0.038 | **0.856** | 0.043 | 0.114 |
| Chase proneness to birds | -0.114 | 0.062 | **0.855** | 0.006 | 0.091 |
| Chase proneness to falling leaves | 0.067 | 0.067 | **0.797** | 0.090 | 0.020 |
| Reactivity to movement of hands | 0.264 | -0.056 | 0.300 | **0.578** | -0.174 |
| Reactivity to clattering dishes | 0.028 | -0.093 | 0.110 | **0.810** | 0.086 |
| Reactivity to phone ringing | -0.038 | 0.122 | -0.160 | **0.700** | 0.319 |
| Avoidance of aversive place | 0.240 | -0.200 | 0.061 | 0.093 | **0.802** |
| Avoidance of examination table | 0.197 | -0.071 | 0.178 | 0.114 | **0.805** |
| Eigenvalue | 2.885 | 2.755 | 2.254 | 1.318 | 1.001 |
| Contribution ratio (%) | 20.6 | 19.7 | 16.1 | 9.4 | 7.2 |
| Cronbach's α | 0.769 | 0.925 | 0.801 | 0.531 | 0.756 |

The questionnaire items for which the absolute loading on a factor was 0.4 or more are shown in boldface.

**Table S1J:** Factor analysis of behavioral items on Labrador Retriever (n = 168)

|  | Factor 1 | Factor 2 | Factor 3 | Factor 4 | Factor 5 |
| --- | --- | --- | --- | --- | --- |
| Sociability with men | **0.948** | 0.039 | 0.028 | -0.012 | 0.062 |
| Sociability with women | **0.947** | 0.035 | 0.042 | 0.007 | 0.067 |
| Sociability with children | **0.820** | -0.015 | -0.009 | 0.052 | 0.240 |
| Fear of engine noises | 0.007 | **0.818** | 0.243 | 0.051 | -0.048 |
| Fear of thunder | 0.037 | **0.844** | -0.086 | 0.100 | 0.079 |
| Fear of heavy traffic | 0.007 | **0.880** | 0.104 | 0.111 | 0.128 |
| Chase proneness to cats | 0.047 | 0.200 | 0.072 | 0.017 | **0.858** |
| Chase proneness to birds | 0.202 | 0.048 | 0.203 | 0.075 | **0.867** |
| Chase proneness to falling leaves | 0.218 | -0.154 | 0.348 | 0.134 | **0.553** |
| Reactivity to movement of hands | 0.067 | 0.091 | **0.574** | 0.064 | 0.294 |
| Reactivity to clattering dishes | -0.016 | 0.097 | **0.592** | 0.132 | 0.258 |
| Reactivity to phone ringing | -0.010 | 0.055 | **0.843** | -0.065 | -0.044 |
| Avoidance of aversive place | 0.092 | 0.270 | -0.004 | **0.739** | 0.124 |
| Avoidance of examination table | -0.046 | -0.004 | 0.104 | **0.855** | 0.024 |
| Eigenvalue | 3.556 | 2.409 | 1.775 | 1.180 | 1.032 |
| Contribution ratio (%) | 25.4 | 17.2 | 12.7 | 8.4 | 7.4 |
| Cronbach's α | 0.904 | 0.780 | 0.517 | 0.511 | 0.759 |

The questionnaire items for which the absolute loading on a factor was 0.4 or more are shown in boldface.

**Table S1K:** Factor analysis of behavioral items on French Bulldog (n = 202)

|  | Factor 1 | Factor 2 | Factor 3 | Factor 4 | Factor 5 |
| --- | --- | --- | --- | --- | --- |
| Sociability with men | 0.007 | **0.950** | -0.014 | 0.000 | 0.005 |
| Sociability with women | -0.013 | **0.948** | -0.038 | 0.032 | 0.077 |
| Sociability with children | 0.099 | **0.845** | -0.064 | -0.039 | 0.104 |
| Fear of engine noises | -0.008 | 0.064 | **0.804** | -0.044 | 0.128 |
| Fear of thunder | 0.085 | -0.163 | **0.768** | -0.007 | 0.029 |
| Fear of heavy traffic | -0.009 | -0.010 | **0.867** | 0.135 | 0.023 |
| Chase proneness to cats | **0.855** | 0.098 | -0.003 | -0.064 | 0.005 |
| Chase proneness to birds | **0.893** | 0.076 | 0.091 | -0.032 | 0.135 |
| Chase proneness to falling leaves | **0.741** | -0.080 | -0.011 | 0.190 | 0.126 |
| Reactivity to movement of hands | 0.174 | 0.215 | 0.070 | 0.227 | **0.600** |
| Reactivity to clattering dishes | 0.084 | -0.024 | 0.022 | 0.233 | **0.745** |
| Reactivity to phone ringing | 0.031 | 0.039 | 0.102 | -0.111 | **0.833** |
| Avoidance of aversive place | 0.028 | -0.003 | 0.050 | **0.871** | 0.075 |
| Avoidance of examination table | 0.036 | -0.007 | 0.015 | **0.856** | 0.162 |
| Eigenvalue | 2.935 | 2.518 | 1.862 | 1.684 | 1.161 |
| Contribution ratio (%) | 21.0 | 18.0 | 13.3 | 12.0 | 8.3 |
| Cronbach's α | 0.781 | 0.907 | 0.750 | 0.719 | 0.613 |

The questionnaire items for which the absolute loading on a factor was 0.4 or more are shown in boldface.

**Table S1L:** Factor analysis of behavioral items on Pomeranian (n = 152)

|  | Factor 1 | Factor 2 | Factor 3 | Factor 4 | Factor 5 |
| --- | --- | --- | --- | --- | --- |
| Sociability with men | **0.958** | -0.009 | 0.142 | 0.049 | -0.010 |
| Sociability with women | **0.951** | 0.001 | 0.136 | 0.087 | -0.052 |
| Sociability with children | **0.887** | -0.097 | 0.164 | 0.044 | -0.053 |
| Fear of engine noises | -0.051 | **0.812** | 0.120 | 0.100 | 0.196 |
| Fear of thunder | -0.045 | **0.774** | -0.223 | 0.151 | 0.024 |
| Fear of heavy traffic | -0.010 | **0.898** | 0.023 | 0.054 | 0.145 |
| Chase proneness to cats | 0.145 | -0.100 | **0.773** | 0.098 | 0.055 |
| Chase proneness to birds | 0.126 | 0.053 | **0.826** | 0.054 | 0.100 |
| Chase proneness to falling leaves | 0.120 | -0.010 | **0.716** | 0.157 | 0.012 |
| Reactivity to movement of hands | 0.125 | 0.210 | 0.219 | **0.649** | 0.031 |
| Reactivity to clattering dishes | -0.021 | 0.057 | 0.122 | **0.792** | 0.177 |
| Reactivity to phone ringing | 0.069 | 0.048 | 0.013 | **0.795** | 0.117 |
| Avoidance of aversive place | -0.064 | 0.216 | 0.080 | 0.208 | **0.841** |
| Avoidance of examination table | -0.040 | 0.122 | 0.078 | 0.119 | **0.904** |
| Eigenvalue | 3.411 | 3.043 | 1.659 | 1.199 | 1.050 |
| Contribution ratio (%) | 24.4 | 21.7 | 11.9 | 8.6 | 7.5 |
| Cronbach's α | 0.936 | 0.763 | 0.749 | 0.656 | 0.793 |

The questionnaire items for which the absolute loading on a factor was 0.4 or more are shown in boldface.

**Table S1M:** Factor analysis of behavioral items on Cavelier King Charles Spaniel (n = 160)

|  | Factor 1 | Factor 2 | Factor 3 | Factor 4 | Factor 5 |
| --- | --- | --- | --- | --- | --- |
| Sociability with men | **0.909** | -0.054 | 0.076 | -0.020 | -0.093 |
| Sociability with women | **0.924** | -0.011 | 0.113 | 0.031 | -0.077 |
| Sociability with children | **0.873** | 0.060 | 0.070 | 0.072 | -0.066 |
| Fear of engine noises | -0.053 | **0.743** | -0.048 | -0.044 | 0.148 |
| Fear of thunder | 0.024 | **0.778** | 0.076 | -0.048 | 0.141 |
| Fear of heavy traffic | 0.030 | **0.886** | -0.008 | 0.077 | 0.076 |
| Chase proneness to cats | -0.042 | 0.054 | **0.838** | 0.092 | -0.083 |
| Chase proneness to birds | 0.134 | -0.011 | **0.901** | 0.057 | 0.056 |
| Chase proneness to falling leaves | 0.210 | -0.028 | **0.742** | 0.202 | 0.205 |
| Reactivity to movement of hands | 0.203 | -0.210 | 0.131 | **0.559** | 0.299 |
| Reactivity to clattering dishes | -0.051 | 0.312 | 0.243 | **0.701** | 0.027 |
| Reactivity to phone ringing | -0.007 | -0.091 | 0.035 | **0.841** | -0.067 |
| Avoidance of aversive place | -0.265 | 0.189 | 0.004 | -0.018 | **0.799** |
| Avoidance of examination table | -0.015 | 0.214 | 0.099 | 0.100 | **0.820** |
| Eigenvalue | 3.105 | 2.722 | 1.951 | 1.249 | 1.081 |
| Contribution ratio (%) | 22.2 | 19.4 | 13.9 | 8.9 | 7.7 |
| Cronbach's α | 0.912 | 0.731 | 0.805 | 0.589 | 0.688 |

The questionnaire items for which the absolute loading on a factor was 0.4 or more are shown in boldface.

**Table S1N: Factor analysis of behavioral items on Shih Tzu (n = 145)**

|  | Factor 1 | Factor 2 | Factor 3 | Factor 4 | Factor 5 |
| --- | --- | --- | --- | --- | --- |
| Sociability with men | 0.011 | **0.925** | 0.102 | -0.068 | -0.031 |
| Sociability with women | -0.007 | **0.957** | 0.089 | 0.026 | 0.013 |
| Sociability with children | -0.018 | **0.852** | 0.103 | 0.103 | 0.067 |
| Fear of engine noises | **0.740** | 0.010 | -0.009 | 0.011 | 0.309 |
| Fear of thunder | **0.694** | -0.054 | -0.026 | -0.025 | 0.040 |
| Fear of heavy traffic | **0.849** | 0.078 | 0.017 | 0.045 | 0.130 |
| Chase proneness to cats | 0.086 | 0.117 | **0.816** | 0.053 | 0.114 |
| Chase proneness to birds | 0.064 | 0.053 | **0.873** | 0.055 | -0.050 |
| Chase proneness to falling leaves | 0.017 | 0.309 | **0.589** | -0.183 | 0.395 |
| Reactivity to movement of hands | 0.187 | -0.001 | 0.114 | 0.184 | **0.817** |
| Reactivity to clattering dishes | -0.026 | 0.046 | 0.028 | **0.752** | 0.354 |
| Reactivity to phone ringing | 0.144 | 0.023 | 0.012 | **0.827** | -0.072 |
| Avoidance of aversive place | **0.608** | -0.154 | 0.357 | 0.325 | -0.169 |
| Avoidance of examination table | **0.617** | 0.064 | 0.385 | 0.130 | -0.246 |
| Eigenvalue | 3.383 | 2.683 | 1.492 | 1.356 | 1.037 |
| Contribution ratio (%) | 24.2 | 19.2 | 10.7 | 9.7 | 7.4 |
| Cronbach's α | 0.757 | 0.907 | 0.738 | 0.474 | - |

The questionnaire items for which the absolute loading on a factor was 0.4 or more are shown in boldface.

**Table S1O:** Factor analysis of behavioral items on Jack Russell Terrier (n = 109)

|  | Factor 1 | Factor 2 | Factor 3 | Factor 4 | Factor 5 |
| --- | --- | --- | --- | --- | --- |
| Sociability with men | **0.938** | 0.038 | -0.045 | -0.024 | 0.001 |
| Sociability with women | **0.952** | 0.042 | -0.059 | 0.032 | -0.022 |
| Sociability with children | **0.825** | 0.260 | -0.083 | 0.042 | -0.087 |
| Fear of engine noises | -0.056 | 0.091 | **0.846** | 0.137 | 0.062 |
| Fear of thunder | -0.049 | -0.286 | **0.656** | -0.096 | 0.258 |
| Fear of heavy traffic | -0.071 | 0.068 | **0.873** | 0.089 | 0.055 |
| Chase proneness to cats | 0.137 | **0.692** | -0.102 | 0.094 | 0.313 |
| Chase proneness to birds | -0.013 | **0.854** | -0.020 | 0.039 | 0.123 |
| Chase proneness to falling leaves | 0.206 | **0.787** | 0.086 | 0.064 | -0.052 |
| Reactivity to movement of hands | 0.065 | 0.183 | 0.078 | **0.748** | 0.023 |
| Reactivity to clattering dishes | 0.182 | 0.064 | 0.019 | **0.717** | 0.374 |
| Reactivity to phone ringing | -0.144 | -0.051 | 0.053 | **0.811** | 0.002 |
| Avoidance of aversive place | -0.118 | 0.226 | 0.198 | 0.138 | **0.764** |
| Avoidance of examination table | -0.015 | 0.062 | 0.112 | 0.089 | **0.866** |
| Eigenvalue | 3.099 | 2.868 | 1.679 | 1.435 | 1.100 |
| Contribution ratio (%) | 22.1 | 20.5 | 12.0 | 10.2 | 7.9 |
| Cronbach's α | 0.915 | 0.725 | 0.702 | 0.651 | 0.671 |

The questionnaire items for which the absolute loading on a factor was 0.4 or more are shown in boldface.

**Table S1P:** Factor analysis of behavioral items on Maltese (n = 84)

|  | Factor 1 | Factor 2 | Factor 3 | Factor 4 | Factor 5 |
| --- | --- | --- | --- | --- | --- |
| Sociability with men | **0.898** | 0.176 | -0.142 | 0.134 | 0.007 |
| Sociability with women | **0.924** | 0.024 | -0.099 | 0.204 | -0.010 |
| Sociability with children | **0.885** | 0.026 | 0.051 | 0.108 | -0.021 |
| Fear of engine noises | -0.121 | 0.034 | **0.603** | 0.000 | 0.312 |
| Fear of thunder | 0.035 | -0.021 | **0.865** | -0.024 | -0.011 |
| Fear of heavy traffic | -0.090 | 0.102 | **0.873** | 0.097 | -0.053 |
| Chase proneness to cats | 0.132 | -0.040 | 0.101 | **0.840** | 0.219 |
| Chase proneness to birds | 0.120 | 0.019 | 0.015 | **0.880** | 0.111 |
| Chase proneness to falling leaves | 0.185 | 0.270 | -0.039 | **0.650** | -0.096 |
| Reactivity to movement of hands | 0.068 | **0.603** | -0.070 | 0.101 | 0.096 |
| Reactivity to clattering dishes | 0.148 | **0.699** | 0.161 | 0.147 | 0.270 |
| Reactivity to phone ringing | -0.015 | **0.838** | 0.075 | -0.056 | -0.006 |
| Avoidance of aversive place | 0.015 | 0.168 | 0.118 | 0.082 | **0.878** |
| Avoidance of examination table | -0.033 | 0.117 | 0.009 | 0.115 | **0.882** |
| Eigenvalue | 3.369 | 2.527 | 1.587 | 1.456 | 1.144 |
| Contribution ratio (%) | 24.1 | 18.1 | 11.3 | 10.4 | 8.2 |
| Cronbach's α | 0.919 | 0.527 | 0.720 | 0.740 | 0.761 |

The questionnaire items for which the absolute loading on a factor was 0.4 or more are shown in boldface.

**Table S1Q: Factor analysis of behavioral items on Pug (n = 95)**

|  | Factor 1 | Factor 2 | Factor 3 | Factor 4 |
| --- | --- | --- | --- | --- |
| Sociability with men | **0.888** | -0.049 | 0.007 | 0.226 |
| Sociability with women | **0.847** | -0.053 | 0.041 | 0.284 |
| Sociability with children | **0.829** | -0.116 | 0.088 | 0.242 |
| Fear of engine noises | 0.060 | 0.012 | **0.758** | 0.033 |
| Fear of thunder | -0.113 | 0.156 | **0.791** | 0.062 |
| Fear of heavy traffic | 0.204 | 0.307 | **0.790** | 0.017 |
| Chase proneness to cats | 0.200 | 0.213 | 0.043 | **0.830** |
| Chase proneness to birds | 0.132 | 0.167 | 0.017 | **0.868** |
| Chase proneness to falling leaves | 0.335 | 0.170 | 0.144 | **0.709** |
| Reactivity to movement of hands | 0.039 | **0.749** | 0.147 | 0.185 |
| Reactivity to clattering dishes | -0.022 | **0.787** | 0.089 | 0.170 |
| Reactivity to phone ringing | **-0.436** | **0.651** | 0.123 | 0.079 |
| Avoidance of aversive place | **-0.446** | -0.073 | **0.527** | 0.265 |
| Avoidance of examination table | **-0.517** | 0.006 | 0.358 | 0.448 |
| Eigenvalue | 3.760 | 3.227 | 1.576 | 1.154 |
| Contribution ratio (%) | 26.9 | 23.0 | 11.3 | 8.2 |
| Cronbach's α | 0.744 | 0.680 | 0.671 | 0.854 |

The questionnaire items for which the absolute loading on a factor was 0.4 or more are shown in boldface.
