# Supplementary material for: "Reactivity to Stimuli” Is a Temperamental Factor Contributing to Canine Aggression
Source: PLoS One. 2014 Jun 27;9(6):e100767. doi: 10.1371/journal.pone.0100767 (PMC4074066; doi:10.1371/journal.pone.0100767)
Supplement: Table S2 — Scores of four types of aggression in 14 breeds. (DOC) [file pone.0100767.s006.doc]

**Table S2**: Scores of four types of aggression in 14 breeds

|  | Owner | | Child | | Stranger | | Dog | |
| --- | --- | --- | --- | --- | --- | --- | --- | --- |
| Breed | score | rank | score | rank | score | rank | score | rank |
| Maltese | 1.81 ± 0.10 | 1 | 1.67 ± 0.12 | 7 | 1.82 ± 0.12 | 5 | 2.17 ± 0.13 | 7 |
| Pomeranian | 1.74 ± 0.08 | 2 | 1.73 ± 0.09 | 4 | 1.80 ± 0.09 | 6 | 1.99 ± 0.10 | 9 |
| Shiba Inu | 1.69 ± 0.05 | 3 | 1.69 ± 0.06 | 6 | 1.85 ± 0.06 | 4 | 2.36 ± 0.06 | 2 |
| Chihuahua | 1.66 ± 0.03 | 4 | 2.20 ± 0.05 | 1 | 2.40 ± 0.05 | 1 | 2.47 ± 0.05 | 1 |
| Toy Poodle | 1.65 ± 0.03 | 5 | 1.51 ± 0.03 | 9 | 1.69 ± 0.04 | 8 | 2.00 ± 0.04 | 8 |
| Papillon | 1.60 ± 0.06 | 6 | 1.58 ± 0.07 | 8 | 1.67 ± 0.07 | 9 | 2.29 ± 0.09 | 5 |
| Yorkshire Terrier | 1.51 ± 0.06 | 7 | 1.70 ± 0.08 | 5 | 1.73 ± 0.08 | 7 | 1.99 ± 0.09 | 10 |
| French Bull dog | 1.46 ± 0.06 | 8 | 1.23 ± 0.05 | 11 | 1.33 ± 0.06 | 12 | 1.76 ± 0.09 | 12 |
| Miniature Dachshund | 1.45 ± 0.03 | 9 | 1.77 ± 0.04 | 3 | 2.03 ± 0.04 | 2 | 2.35 ± 0.04 | 3 |
| Jack Russell Terrier | 1.34 ± 0.07 | 10 | 1.29 ± 0.07 | 10 | 1.47 ± 0.08 | 10 | 2.30 ± 0.11 | 4 |
| Miniature Schnauzer | 1.33 ± 0.05 | 11 | 1.95 ± 0.09 | 2 | 2.00 ± 0.09 | 3 | 2.28 ± 0.09 | 6 |
| Cavalier King Charles Spaniel | 1.17 ± 0.04 | 12 | 1.18 ± 0.05 | 12 | 1.29 ± 0.06 | 13 | 1.50 ± 0.07 | 14 |
| Golden Retriever | 1.15 ± 0.03 | 13 | 1.09 ± 0.03 | 14 | 1.24 ± 0.05 | 14 | 1.71 ± 0.07 | 13 |
| Labrador Retriever | 1.08 ± 0.02 | 14 | 1.17 ± 0.04 | 13 | 1.36 ± 0.06 | 11 | 1.77 ± 0.08 | 11 |

Values are average ± SD of aggression scores in each breed and rank among 14 breeds.
