# Supplementary material for: "Reactivity to Stimuli” Is a Temperamental Factor Contributing to Canine Aggression
Source: PLoS One. 2014 Jun 27;9(6):e100767. doi: 10.1371/journal.pone.0100767 (PMC4074066; doi:10.1371/journal.pone.0100767)
Supplement: Table S3 — Points of five temperamental factors in 14 breeds. (DOC) [file pone.0100767.s007.doc]

**Table S3**: Points of five temperamental factors in 14 breeds

|  | reactivity to stimuli | | sociability with humans | | avoidance of aversive events | | chase-proneness | | fear of sounds | |
| --- | --- | --- | --- | --- | --- | --- | --- | --- | --- | --- |
| Breed | point | rank | point | rank | point | rank | point | rank | point | rank |
| Pomeranian | 2.80 ± 0.08 | 1 | 2.44 ± 0.09 | 9 | 2.66 ± 0.10 | 7 | 2.43 ± 0.08 | 4 | 1.93 ± 0.07 | 4 |
| Maltese | 2.62 ± 0.09 | 2 | 2.29 ± 0.12 | 3 | 3.09 ± 0.13 | 12 | 2.37 ± 0.11 | 3 | 2.12 ± 0.10 | 11 |
| Yorkshire Terrier | 2.62 ± 0.07 | 3 | 2.34 ± 0.08 | 5 | 3.22 ± 0.09 | 13 | 2.32 ± 0.08 | 2 | 2.57 ± 0.08 | 14 |
| Papillon | 2.61 ± 0.06 | 4 | 2.42 ± 0.08 | 7 | 2.86 ± 0.09 | 8 | 2.73 ± 0.07 | 10 | 1.94 ± 0.06 | 5 |
| Toy Poodle | 2.54 ± 0.03 | 5 | 2.43 ± 0.04 | 8 | 2.98 ± 0.04 | 9 | 2.58 ± 0.04 | 8 | 2.09 ± 0.03 | 9 |
| Chihuahua | 2.45 ± 0.04 | 6 | 2.01 ± 0.04 | 1 | 3.34 ± 0.05 | 14 | 2.22 ± 0.04 | 1 | 2.52 ± 0.04 | 13 |
| Miniature Dachshund | 2.45 ± 0.03 | 7 | 2.14 ± 0.03 | 2 | 3.09 ± 0.04 | 11 | 2.54 ± 0.04 | 6 | 2.18 ± 0.03 | 12 |
| Jack Russell Terrier | 2.38 ± 0.09 | 8 | 2.64 ± 0.10 | 12 | 2.24 ± 0.11 | 3 | 3.48 ± 0.09 | 14 | 2.09 ± 0.09 | 10 |
| Cavalier King Charles Spaniel | 2.27 ± 0.07 | 9 | 2.80 ± 0.09 | 13 | 2.38 ± 0.10 | 5 | 2.68 ± 0.10 | 9 | 2.00 ± 0.07 | 6 |
| French Bull dog | 2.23 ± 0.07 | 10 | 2.84 ± 0.08 | 14 | 2.22 ± 0.09 | 2 | 2.94 ± 0.08 | 11 | 1.40 ± 0.05 | 1 |
| Miniature Schnauzer | 2.10 ± 0.06 | 11 | 2.45 ± 0.07 | 10 | 2.60 ± 0.09 | 6 | 3.03 ± 0.08 | 12 | 2.07 ± 0.07 | 7 |
| Shiba Inu | 2.04 ± 0.04 | 12 | 2.33 ± 0.05 | 4 | 2.98 ± 0.06 | 10 | 3.28 ± 0.06 | 13 | 2.08 ± 0.05 | 8 |
| Golden Retriever | 1.64 ± 0.05 | 13 | 2.56 ± 0.08 | 11 | 2.04 ± 0.08 | 1 | 2.54 ± 0.07 | 7 | 1.81 ± 0.06 | 3 |
| Labrador Retriever | 1.64 ± 0.05 | 14 | 2.40 ± 0.08 | 6 | 2.25 ± 0.08 | 4 | 2.45 ± 0.08 | 5 | 1.56 ± 0.06 | 2 |

Values are average ± SD of factor points in each breed and rank among 14 breeds.
